# European Cardiovascular Magnetic Resonance (EuroCMR) registry - multi national tesults from 57 centers in 15 countries

**DOI:** 10.1186/1532-429X-15-S1-O96

**Published:** 2013-01-30

**Authors:** Anja Wagner, Oliver Bruder, Massimo Lombardi, Juerg Schwitter, Albert C van Rossum, Guenter Pilz, Detlev Nothnagel, Steffen E Petersen, Eike Nagel, Sanjay K Prasad, Herbert Frank, Thorsten Dill, Steffen Schneider, Heiko Mahrholdt

**Affiliations:** 1 Cardiology, Comprehensive Cardiology, Stamford, CT, USA; 2 Cardiology, Contilia Heart and Vascular Center, Essen, Germany; 3 Cardiology, C.N.R./Regione Toscana "G. Monasterio Foundation", Pisa, Italy; 4 Cardiology, Cardiac MR Centre, University Hospital (CHUV) Lausanne, Lausanne, Switzerland; 5 Cardiology, VU Medical Centre, Amsterdam, Netherlands; 6 Cardiology, Hospital Agatharied, Hausham, Germany; 7 Cardiology, Klinikum Ludwigsburg, Ludwigsburg, Germany; 8 Barts and The London NIHR Biomedical Research Unit, The London Chest Hospital, London, UK; 9 Division of Imaging Sciences, King's College London BHF, London, UK; 10 CMR Unit, Royal Brompton Hospital, London, UK; 11 Internal Medicine, Krankenhaus Benrath, Düsseldorf, Germany; 12 Cardiology, Donauklinikum Tulln, Tulln, Austria; 13 Department of Statistics, Institut für Herzinfarktforschung, Ludwigshafen, Germany; 14 Cardiology, Robert Bosch Medical Center, Stuttgart, Germany

## Background

The EuroCMR registry determined indications, image quality, safety and impact on patient management of clinical routine CMR in a multi-national European setting.

Furthermore, interim analyses of two specific protocols evaluating the prognostic potential of CMR in patients with coronary artery disease (CAD) and hypertrophic cardiomyopathy (HCM) are presented.

## Methods

Multi-center registry with consecutive enrollment of patients in 57 centers in 15 countries [1].

## Results

27,781 patients were enrolled. The most frequent indications were risk stratification in CAD/ischemia (34.2%), workup of cardiomyopathies (32.2%) and assessment of viability (14.6%). Image quality was diagnostic in 98%. Severe complications were rare (0.03%).

In 61.8% CMR findings had an impact on patient management. In 8.7% the final diagnosis changed based on CMR findings (Table 1).

**Table 1:** Impact of CMR on patient management by indication

	Myocarditis/ Cardiomyopathy	Suspected CAD/Ischemia	Viability
All (from n = 27781)	32.2%	34.2%	14.6%
New diagnosis	11.4%	8.1%	5.3%
Therapeutic consequences:			
Change in medication	25.3%	24.3%	33.2%
Invasive procedure	6.9%	23.1%	24.2%
Hospital discharge	10.4%	14.3%	6.9%
Impact on patient management (new diagnosis and/or therapeutic consequence)	55.1%	71.4%	71.5%

Kaplan-Meier survival curves of the interim analyses showed low adverse event rates in patients with suspected CAD with a normal stress CMR (1.0% per year), and in HCM without delayed enhancement (2.7% per year).

## Conclusions

The most important CMR indications in Europe are risk stratification in suspected CAD/ischemia, work-up of cardiomyopathies and assessment of viability. CMR is a safe procedure, has diagnostic image quality in more than 98% of cases, and its results have a strong impact on patient management. Interim analyses underscore the prognostic value of clinical routine CMR in patients with CAD and HCM.

## Funding

Medtronic Inc., Minneapolis MN, USA.

Novartis International AG, Basel, Switzerland.

Siemens Health Care, Erlangen, Germany.
